# Culture circle in primary care: dialogues with managers on health promotion

**DOI:** 10.1590/1980-220X-REEUSP-2023-0420en

**Published:** 2024-07-08

**Authors:** Ivonete Teresinha Schulter Buss Heidemann, Michelle Kuntz Durand, Izaltina Adão, Priscila Juceli Romanoski, Adriana Rufino Moreira, Aline Megumi Arakawa Belaunde, Vladimir Araujo da Silva, Kamila Soares Maciel

**Affiliations:** 1 Universidade Federal de Santa Catarina, Departamento de Enfermagem, Programa de Pós-Graduação em Enfermagem. Florianópolis, SC, Brazil.; 2 Universidade Federal de Santa Catarina, Departamento de Enfermagem. Florianópolis, SC, Brazil.; 3 Universidade Federal de Santa Catarina, Departamento de Saúde Pública, Programa de Pós-Graduação em Saúde Coletiva. Florianópolis, SC, Brazil.; 4 Universidade Federal de Santa Catarina, Departamento de Fonoaudiologia. Florianópolis, SC, Brazil.; 5 Universidade Federal de Santa Catarina, Centro de Ciências Rurais, Departamento de Biociências e Saúde Única. Santa Catarina, SC, Brazil.

**Keywords:** Health Promotion, Unified Health System, Primary Health Care, Community-Based Participatory Research, Social Determinants of Health, Promoción de la Salud, Sistema Único de Salud, Atención Primaria de Salud, Investigación Participativa Basada en la Comunidad, Determinantes Sociales de la Salud

## Abstract

**Objective:**

To identify health promotion strategies used by managers in primary health care.

**Method:**

Qualitative research, of a participant action nature, which adopted the Culture Circle proposed by Paulo Freire as its methodological reference. Eleven primary health care managers from a medium-sized municipality in southern Brazil took part.

**Results:**

Nine generative themes emerged, categorized into four themes that highlight the interconnection between health promotion, social determinants and primary health care. These themes highlight preventive approaches, healthy habits and underline the need for a multidisciplinary approach to health care, recognizing the complexity of the dimensions involved, the influence of social determinants, environmental and health issues. These aspects call for intersectoral policies and actions, demonstrating the viability of health promotion in line with the principles of the Unified Health System.

**Final considerations:**

The autonomy of professionals working in primary health care services is highlighted, especially that of nurses, who play a central role in connecting and organizing health promotion actions.

## RESUMEN

**Objetivo:**

Identificar las estrategias de promoción de la salud utilizadas por gestores en la atención primaria de salud.

**Método:**

Investigación cualitativa, de tipo acción participante, que adoptó como referencia metodológica el Círculo de Cultura propuesto por Paulo Freire. Participaron 11 gestores de atención primaria de salud de un municipio de tamaño medio del sur de Brasil.

**Resultados:**

Surgieron nueve temas generativos, categorizados en cuatro temáticas que destacan la interconexión entre la promoción de la salud, los determinantes sociales y la atención primaria de salud. Estos temas destacan los enfoques preventivos, los hábitos saludables y subrayan la necesidad de un enfoque multidisciplinar de la atención sanitaria, reconociendo la complejidad de las dimensiones implicadas, la influencia de los determinantes sociales y las cuestiones medioambientales y sanitarias. Estos aspectos exigen políticas y acciones intersectoriales, demostrando la viabilidad de la promoción de la salud en consonancia con los principios del Sistema Único de Salud.

**Consideraciones finales:**

Se destaca la autonomía de los profesionales que actúan en los servicios de atención primaria de salud, especialmente la de las enfermeras, que desempeñan un papel central en la conectividad y organización de las acciones de promoción de la salud.

## INTRODUCTION

Health promotion encompasses promising strategies for addressing health issues, both individually and collectively, with the aim of improving health conditions. It is characterized by intra-sectoral and inter-sectoral cooperation, establishes connections with social protection networks and promotes broad participation and comprehensive social control. Its purpose is to reduce vulnerabilities and health risks arising from social, economic, political, cultural and environmental determinants. Considering the comprehensive conception of the health-disease process and its determinants, this strategy seeks to integrate technical and popular knowledge. Health promotion involves actions aimed at changing individual behavior, collective interventions and integrated public policies^(1)^.

The First International Conference on Primary Health Care, held in Alma-Ata in 1978, brought contributions to implement the goal of “health for all by the year 2000”, with emphasis on the proposal of Primary Health Care (PHC) as a central element in the organization of systems aimed at promoting universal health^(2)^. Among the various International Conferences that have discussed Health Promotion, we highlight the one held in 1986 in Ottawa, Canada, when countries committed themselves to taking health-promoting actions in their policies. It recommended five basic strategies for health promotion: building public health policies; creating supportive environments; strengthening community action; developing personal skills; and reorienting health services^(3)^. The 9th Shanghai Conference in 2016 therefore reinforced health promotion activities in line with the new 2030 Agenda and the SDGs^(1)^. The Shanghai Declaration emphasizes the importance of people’s autonomy in controlling their own health and choosing a healthy lifestyle^(4)^. The commitments made aim to accelerate funding for health promotion and the implementation of the SDGs. Several proposals have been put forward to achieve this goal, including the adoption of policies that favor women and populations in social and environmental vulnerability, the recognition of the right to health as a common and fundamental good for all and the emphasis on the importance of cities and communities as essential environments for promoting the health of the population^(1)^.

The Global Conference on Primary Health Care in Astana, 2018 sought to reaffirm the commitment to PHC aimed to Universal Health Coverage and the Sustainable Development Goals (SDGs). However, Universal Health Coverage, by shifting the right to health based on equality to a principle of affordable coverage, goes against the spirit of Alma-Ata and the SDGs, and could result in disparities. Critics of the Astana Charter highlight this discrepancy, advocating for comprehensive PHC and the universal right to global health. However, the Alma-Ata Declaration remains an international benchmark, surpassing the Astana Declaration in principles of social justice and a comprehensive approach to PHC^(5)^.

It should be understood that it is necessary to consider people with hyperconnectivity as part of the vulnerable population. There is an urgent need for public policies to mitigate psychological suffering and thus promote the mental health of the population assisted by us, health professionals, with the aim of achieving comprehensive health care. In line with the Social Determinants of Health (SDH), health promotion subsidizes access to information, broadens people’s life experiences and skills, enabling them to make healthier lifestyle choices. This approach aims to reduce disparities in the population’s state of health, guaranteeing equitable care and resources^(6)^.

By organizing care in the healthcare network, PHC conducts health-promoting actions through intersectoral approaches, encompassing the health, social assistance and education sectors^(6)^. However, in order to achieve equity in health, it is crucial to combat the inequalities resulting from historical and current policies and practices, including the social and structural determinants that result in exposure to food insecurity, housing instability and difficulty in accessing goods, services and opportunities. This challenge is particularly relevant when considering people in conditions or at risk of vulnerability and social exclusion associated with factors such as race, ethnicity, gender, sexual orientation and immigration^(7)^. Given this context, this study aims to identify the health promotion strategies used by PHC managers.

## METHOD

This is a qualitative study, having a participant action nature, adopting the Research Itinerary proposed by Paulo Freire as its theoretical and methodological framework, implemented through Culture Circles. In a dialogical and dialectical process, dynamic, critical and horizontal interactions are promoted between the participants, their experiences, knowledge and skills, with a view to political, social and educational transformation^(8)^. The Research Itinerary covers three dialectical and flexible moments, allowing for the use of inductive or deductive elements in the stages of thematic investigation, coding and decoding, and critical unveiling^(9)^. The writing of this article follows the protocol of the Consolidated Criteria for Reporting Qualitative Research (COREQ)^(10)^.

The Culture Circle took place in a municipality with around 40,000 inhabitants, with a strong agricultural, commercial and industrial sector. We entered the field after contacting a researcher representing a local university, establishing a theoretical-practical connection with PHC. At the time, 11 PHC managers attended the event. This study covers municipalities in the mesoregion of a state in southern Brazil, providing relevant results through the application of the research method focused on the theme of health promotion. The inclusion criteria considered the status of manager and consent to actively participate in the culture circle. Managers away on vacation or leave during the thematic investigation were excluded. To preserve the anonymity of the participants, flower codenames chosen by them were used.

Data collection happened during a two-hour Culture Circle on April 11, 2023. This meeting took place in the auditorium of the Municipal Health Department, coinciding with the monthly meeting of municipal managers. During the general meeting, a two-hour period was set aside for the Culture Circle, as previously agreed with the participants. The mediators responsible for facilitating the Culture Circle were experienced, specialized researchers with in-depth knowledge of Paulo Freire’s Research Itinerary, used in this qualitative study. It is important to note that the mediators remained neutral, with no ties to the participants, creating an environment conducive to dialog, preserving ethics and anonymity among those involved.

On the day of the Culture Circle, the objectives of the study and the methodology to be adopted were initially presented. The participants then took part in a dynamic presentation and filled in socio-economic data. This was followed by the initial phase of the study, known as thematic research. Within the context of Paulo Freire’s Research Itinerary, it is not common to refer explicitly to a “codebook” or “script” in the traditional sense, since this method favors the collective construction of knowledge and flexibility in the research process.

The participants were divided into three sub-groups, in which they were encouraged to discuss and record the generating themes on colored cards, answering the guiding question: “What health promotion strategies are addressed in PHC? Identify the potential and challenges associated with working in PHC.” After the research stage, the subgroups met again in a large group and shared their thematic findings. Nine generating themes were identified and subsequently grouped into four themes of significant relevance: 1) Health education: health promotion through health education; health education groups; the Health in the School Program: health promotion strategies/practices; Integrative and Complementary Health Practices. 2) The relationship between the SDH and the health-disease situation: SDH and continuing education; SDH and the health-disease situation of “users”. 3) Principles of the Unified Health System (SUS) and intersectorality. 4) Connectivity and the relationship with mental health: technological dependence and connectivity; social networks and mental health promotion.

**Figure 1:**
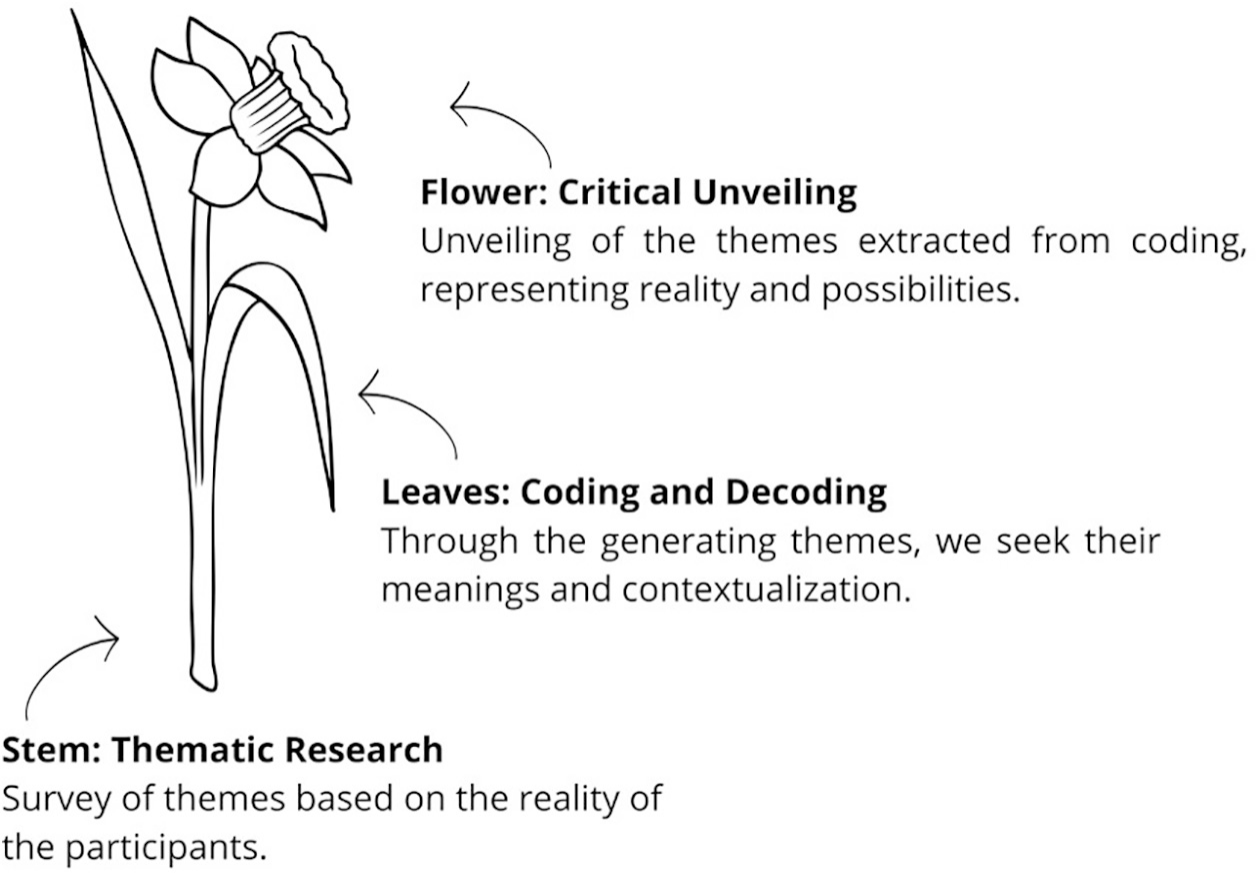
Research Itinerary: analogy with the flower.

Source: prepared by the authors.

To encourage dialogue and make it more dynamic, interactive and concrete, the participants chose the analogy of a flower to represent the stages of Paulo Freire’s Research Itinerary and the context of their work as managers in a city in the Midwest of Santa Catarina state, as shown in Figure 1. This analogy reflects on the interconnected parts of a flower, analogous to Paulo Freire’s Research Itinerary.

In the second stage, called coding and decoding, the four themes that emerged in the main group were discussed. The mediators engaged in dialogues with the participants, encouraging them to contemplate their reality and reflect on health promotion and the SDH.

In the critical unveiling stage, the discussion of the themes outlined in the previous phases was deepened. With the participants’ consent, the dialogues were recorded for approximately four hours using audio recorders to capture the discussions. The main themes addressed were recorded in a field notebook and later transcribed for detailed analysis.

Data analysis was conducted simultaneously with the development of the Culture Circle, in line with the principles of Paulo Freire’s Research Itinerary^(9)^. This continuous analytical process was carried out in interaction with all the study participants^(8)^, managers and mediators, without the need to use software to support qualitative data analysis.

In order to comply with the ethical aspects inherent in research with human beings, the researchers followed the recommendations of Resolution 466/12 of the National Health Council and its complementary resolutions^(11)^.

## RESULTS

The analysis explored and categorized nine generating themes into four themes: 1) Health education; 2) Relationship between SDH and the health-disease situation; 3) SUS principles and intersectorality; 4) Connectivity and the relationship with mental health.

### 1. Health Education

Health education was widely discussed, and the participants were unanimous in considering it a key component in health promotion. The debate focused on the importance of health guidelines and the need to raise public awareness of health education. Actions aimed at health promotion, disease prevention and the importance of involving support groups and the community in general in the dissemination of health-related information were addressed. The importance of a multidisciplinary approach was highlighted, recognizing that a single professional cannot cover all facets of health.

*Multi-professional teams are important for helping the community and strengthening health as a whole. Our work seeks collectivity and organization in order to offer quality care in each unit* (Violet).

They highlighted the principle of comprehensiveness as one of the municipality’s strengths, emphasizing that *this principle guides our approach to reaching different audiences through different strategies* (Lotus Flower).

The therapeutic groups cover various health conditions, such as pregnant women, hypertensive patients, diabetics and smokers. On the other hand, the Health in the School Program establishes partnerships with the education department and promotes interventions from early childhood to high school, addressing various themes. *One example is the ‘Healthy Life’ project, the result of a partnership between the health and sports departments, which offers dance classes for different age groups and promotes not only physical health, but also the satisfaction and well-being of the participants* (Violeta).

Another key point highlighted is the influence of culture on health promotion. Culture plays a fundamental role in shaping lifestyle habits, attitudes towards health and the way people seek health care. They mentioned in *many cultures, the main focus can be on curative medical care, i.e. treating illnesses after they have manifested, rather than prioritizing prevention* (Tulip).

*The emphasis on the curative part can be a reflection of a community’s cultural beliefs and values. Seeking medical care can be seen as a demonstration of care and responsibility, while health promotion, which often focuses on prevention, may be less emphasized* (Orchid).

Health promotion was also discussed in the group, in the sense of understanding that integrative and complementary health practices are important for the implantation and implementation of health promotion in the territory.

*Today we have auriculotherapy, reiki, and we have colleagues here who are therapists and who try to associate this with health promotion. Today we know that this search for the relationship between health promotion and the spiritual area, the concepts of directed speech, of a warm welcome, of an environment where health promotion can be worked on* (Herb of Grace).

### 2. Relationship Between the SDH and the Health-Disease Situation

The participants recognized the impact of SDHs that are not under the direct control of health professionals, such as environmental issues, pollution from companies and poor sanitary conditions. These factors can influence the emergence of diseases and impact on the well-being of the community, but they go beyond the management of health units, depending on actions and measures at municipal, state and national level. The participants emphasized that *health professionals can play a role in preventing and treating diseases, but resolving these issues requires collective efforts at higher levels* (Rose).

They discussed the importance of educating the population about social determinants that can be raised awareness, such as lifestyle and hygiene conditions. They addressed the challenges faced in effective communication and understanding in health interventions. They also reflected on the need to train health professionals and the time constraints for health promotion, given the daily demands that tend to hinder the necessary coverage.

### 3. SUS Principles and Intersectorality

PHC professionals highlighted the applicability of health promotion anchored in the principles of the SUS: universality, equity, comprehensiveness, decentralization and population participation. They highlighted the implementation of actions aimed at groups, emphasizing the importance of population stratification for more effective health care, with a focus on chronic conditions.

*When we talk about promotion, we try to reach all audiences in different ways. [...] each unit works with its own team, its own area, but we always try to include groups of pregnant women, hypertensive patients, diabetics, smokers, insulin addicts*... (Lotus Flower).

The intersectoral nature of the statements highlights the need for professionals from different areas and departments to be involved in order for health promotion actions to be effective.

*According to the calendar, we work on all the themes and axes. Sometimes we also deviate a little, as requested by the secretariat, when they think it’s necessary to address a topic that is more relevant at the time. [...] the ‘Healthy Life’ project works in the neighborhoods! It’s a partnership between the sports department and the health department, which offers dance classes for all age levels. It has a very wide reach and very good participation!”* (Lotus flower).

In this context, the importance of the autonomy of professionals is highlighted, especially nurses, who play a central role in coordinating and integrating health promotion actions, *... in organizing the demand from the units, so as to be able to serve our public in the best possible way* (Flor de Lótus).

### 4. Connectivity and the Relationship with Mental Health

Technology was highlighted as a determinant to be considered when assessing the causes of mental illness in all age groups, alerting us to the lack of clinical protocols to help deal with the new emerging demands.

*Today, we have not only adolescents, but also adults and the elderly who don’t have much to do, who are on social networks receiving information that is causing a great deal of psychological distress, a great deal of dependency. And we don’t know how to manage these people* (Herb of Grace).

The professionals showed knowledge and concern about their preparation to deal with illnesses resulting from excessive exposure to technology, prolonged time in front of screens and children’s early exposure to connectivity. These factors can cause damage not only to users’ mental health, but also to family and social relationships. This highlights the importance of educating and raising awareness among the population about these issues.

*We’re going to have to know how to handle it, how we’re going to welcome and not only welcome the person with technological dependency, but also know how to welcome the family. [...] dependency is so high... Most people here have children. You go to a restaurant, the child is crying, the mother is on her cell phone and the child is eating and watching a movie* (Herb of Grace).

They also expressed concern about the lack of public policies aimed at advertising and publicity on social networks, especially with regard to health promotion actions, with a focus on mental health.

*We are living through a very large pandemic of drug addiction [...] if we look at the TV today, how many commercials do you see talking about drugs? None! You only see beer commercials! We need to better understand what crack is and you don’t see that either!”* (Herb of Grace).

Through dialogues with the participants, a strong relationship was identified between connectivity and an experienced vulnerability, associated with increased anxiety and stress, as well as the difficulty of connecting with technological resources.

## DISCUSSION

The results show that the health promotion strategies employed by PHC managers cover, or indicate a concern to cover, the five strategies recommended in the Ottawa Charter^(3)^. In addition to highlighting health education as a fundamental element, they also identify issues that can contribute to the implementation of strategies that have not been addressed, promoting equity in health^(7)^ and achieving the SDGs^(12)^ which are central to global health and international development.

### 1. Health Education

Health education is seen as one of the health promotion strategies, representing broad approaches aimed at improving individual and collective living conditions. In addition to clinical considerations, it encompasses broader disease prevention actions, the promotion of a healthy lifestyle, with an emphasis on physical activity, a balanced diet, stress reduction, mental health promotion, access to health services and health education, among others^(1,2)^.

Through guidance and raising awareness among the population, there is an opportunity to broaden students’ knowledge and understanding of their own reality^(13)^. By targeting specific groups, health education interventions have the potential to bring about significant changes in the level of knowledge and attitudes, favoring the modification of habits and behaviors^(14)^. In this context, health education is considered one of the main strategic axes for health promotion, with the ability to transform people’s living conditions and health^(15)^. In fact, this empowerment and protagonism, inherent to health education, represent the strategy for “strengthening community action” in PHC, as recommended by the Ottawa Charter^(3)^. promoting autonomy and the sharing of experiences in search of quality of life^(15)^. However, in order to achieve impacts on the individual, social and environmental dimensions, it is crucial to involve a multidisciplinary team^(14)^.

The work of professionals goes beyond guidance and passing on information, and it is essential to incorporate interdisciplinary approaches, planning, content and methods that are appropriate to the local reality. In fact, the “Healthy Life” Project, mentioned by managers and drawn up on the basis of the aforementioned assumptions, is in line with the “creating supportive environments” strategy recommended in the Ottawa Charter^(3)^. This approach makes it possible to reorient and integrate health care. It is imperative that this education be based on the principles proposed by Paulo Freire, characterized as a critical insertion into reality, permeated by dialogue, reflection, communication, collaborative practices and reciprocity between those involved. This leads to the construction of new knowledge and the transformation of both the individual and the world^(16)^.

When it comes to intersectorality, the Health in the School Program is a significant health promotion strategy capable of expanding intersectoral partnerships and offering interventions adapted to different levels of schooling, covering various themes suited to the local reality. In fact, the program strengthens the relationship between the education and health networks, but it took many years before professionals from both areas were able to fully understand the importance of intersectoral action^(17)^. By bringing health services closer to schools, the Health in the School Program allows for a more comprehensive intervention, contributing directly to the formation of more aware and healthier people.

We can also see the significant potential of Integrated and Complementary Practices as a promising strategy for health promotion and comprehensive care. Currently, the National Program for Integrated and Complementary Practices recognizes 29 types of therapies based on non-conventional health knowledge and practices^(18)^, which have gained increasing popularity due to their benefits, beliefs and values. This recognition converges with the influence of culture on health promotion, since it enables a deeper understanding of habits, attitudes and the way people seek health care.

### 2. Relationship Between the SDH and the Health-Disease Situation

The expanded conception of the health-disease process is based on the assumption that the SDH – human conditions related to food, sanitation, housing, work, income, access to education and health services, transportation, a stable ecosystem and sustainable resources – influence the degree of susceptibility to risk factors and the occurrence of health problems in the population^(19)^. In fact, a study carried out with 27 health professionals, including 18 educators, identified that unfavorable living conditions, unhealthy habits and fragile social relationships tend to negatively influence the regular monitoring of growth and development, as well as the school performance of children^(20)^.

Based on this concept, the professionals stressed the importance of raising awareness among the population about adopting a healthy lifestyle and providing guidance on hygiene. During the Culture Circle, they emphasized behavioral, environmental/health, economic and social factors, to the detriment of the psychological factors inherent in the health-disease situation.

Nevertheless, it is essential to consider the interaction of personal, community and social forces in preserving individual and collective environmental and health conditions. Accepting that the health situation is determined implies underestimating the potential of nature, creativity and autonomy of people and society, and their ability to self-organize, act and modify or deconstruct what has been historically constructed by human action^(21)^.

Perhaps because of their lack of clarity regarding the importance of considering the above-mentioned aspects, professionals tend to believe that environmental issues, such as industrial pollution and poor sanitary conditions, fall outside their management scope. However, the managers’ perception that resolving these issues requires collective efforts at higher levels, especially the criticism of the lack of publicity and advertising on TV for tackling illicit drug use and promoting mental health, suggests and/or could support the “building public health policies” strategy recommended in the Ottawa Charter^(3)^.

### 3. SUS Principles and Intersectorality

The SDH are conditions that shape the process of health and disease. Currently, in addition to the proximal determinants related to lifestyle and the distal determinants of income, education, occupation, family structure, availability of health services, social support, racial discrimination, which were already known, it is necessary to reflect on environmental factors, digital health, migration, commercial determinants, among others^(22)^. In our study, professionals recognized the impacts of SDH and stressed that education has a good prospect when dealing with chronic conditions, providing global information and support for self-care.

A study using data from the 2013 National Health Survey, considering socioeconomic inequalities in Brazil and lifestyle as determinants of health, highlighted the need to monitor these disparities in order to guide public policies. It found that inequalities are more prevalent among people with less schooling, who are non-white and do not have private health insurance. Males have higher rates of smoking, alcohol consumption and fatty meats, with a lower intake of fruit and vegetables^(23)^.

Worldwide, the reduction of Chronic Non-Communicable Conditions (NCDs) is essential in the context of global development, in line with the SDG target of reducing premature mortality by a third by 2030. This study highlights the interconnection of these targets with nine other goals, emphasizing the need for strategies for economic gains and the planning of sustainable cities^(24)^. The highest risks of mortality from NCDs are observed in low- and middle-income countries, emphasizing the importance of intersectoral approaches that cover goals such as poverty reduction, combating hunger, education, gender equality, economic growth, equity, life in cities and sustainable production and consumption^(24)^.

A study in England (2016–2017) regarding children aged 10 to 11, found a prevalence of obesity of 26% in more deprived regions while it was 11% in less deprived regions. It is noteworthy that the inequality in childhood obesity persists into adulthood, resulting in a growing disparity in the health problems associated with obesity^(25)^.

The climate emergency, the epidemic of non-communicable diseases and the significant impact of specific sectors (tobacco, ultra-processed foods, fossil fuels and alcohol) reveal the magnitude and high economic costs associated with these challenges^(26)^. A study that sought to define and conceptualize the commercial determinants of health points out that, although commercial determinants can have positive impacts, there is substantial evidence that certain practices and products, especially those of large transnational companies, contribute to health problems, environmental damage and avoidable social and health inequalities. Commercial determinants need special attention, as they often ignore public policies in favor of profit, weakening governments and the state. This study also emphasizes the urgency of implementing effective food regulations by the state^(26)^.

The SDH shape four significant behavioral risk factors: inadequate diet, lack of physical activity, smoking and excessive alcohol consumption, as well as three pathological conditions – hypertension, diabetes and obesity^(25)^. Our research shows the daily presence of educational practices in groups, promoting the principles of the SUS.

Comprehensiveness, understood as the range of services that PHC can offer to the citizens, is the essence of PHC and is the central axis for the operationalization of the other attributes. However, an evaluation of the comprehensiveness of PHC in different countries, carried out by means of a systematic review using the Primary Care Assessment Tool (PCATool), indicates that the low degree of orientation of PHC towards the comprehensiveness of the services available can be interpreted as a lack of understanding of the real demands of users, requiring concrete actions to strengthen PHC as the basis of health systems^(27)^.

With regard to intersectoriality, the “Healthy Life” Project, presented by the managers, incorporates intersectoriality by uniting the health and sports departments, stratifying the population into groups such as pregnant women, hypertensive patients, diabetics and smokers, following the strategy of “reorienting health services” recommended in the Ottawa Charter^(3)^. However, the criticism of the culture centered on curative medical care is not accompanied by concrete proposals to tackle the problem identified. In addition, the lack of mention of specific strategies for vulnerable groups^(7)^ suggests resistance or difficulty on the part of managers in dealing with these groups, jeopardizing the achievement of equity in health.

### 4. Connectivity and the Relationship with Mental Health

In our research, we also highlighted concerns about connectivity and its relationship with mental health, a determinant that has been increasingly studied in the context of chronic conditions. According to the WHO, mental health conditions account for 16% of the global burden of disease and injury in people aged 10 to 19 years. Depression is one of the main causes of illness and disability among adolescents, while suicide is the third leading cause of death in this age group (15 to 19 years). The repercussions of neglecting adolescents’ mental health extend beyond adolescence, adversely affecting physical and mental health and restricting future opportunities. The report also highlights that the influence of the media and gender norms can amplify the disparity between the reality experienced by an adolescent and their perceptions or aspirations for the future^(28)^.

In contemporary times, we face an avalanche of information driven by the use of the internet and social networks. Although these tools facilitate various daily activities, their constant presence can have adverse effects, diverting our attention and focus when we are with significant people, such as our family members. This global phenomenon prompts reflection on the appropriate management of hyperconnectivity, considering its impact on mental health. Hyperconnectivity^(29)^ encompasses all forms of communication on the web, involving interactions between people, from person to machine and between machines. It refers to the constant connection to technological devices such as smartphones, notebooks and computers.

On the web, we can reinvent ourselves; in this world where we value HAVING more than BEING, we subject ourselves to self-coercion that can trigger transformations in the way we live and suffer. On social media, we present what we want to be, not necessarily who we really are. The digital revolution, with its acute hyperconnection, raises relevant discussions about our freedom of expression, subjecting us to a dictatorship of the best performance, where we only show the positive side and, if necessary, invent it. In this context, it is crucial to consider the impacts on psychic and narcissistic suffering, as new dynamics emerge in love relationships, work and personal interactions, altering the psyche in the face of this new reality^(30)^.

One of the main concerns of professionals, as shown in the study, is the management of the numerous information provided by technology to users. The gap in the continuing training of professionals and the subsequent difficulty in dealing with the new emerging demands, as pointed out by managers, highlight the importance and the lack of implementation of the “developing personal skills” strategy recommended in the Ottawa Charter^(3)^.

One of the limitations of this study was the challenge of finding a convenient time for all the professionals and managers to take part in the Culture Circle, considering the load of activities inherent in their work process.

## Final Considerations

This study has shown that the health promotion strategies adopted by PHC managers are connected to the five strategies recommended in the Ottawa Charter or can help achieve the strategies not covered, health equity and the SDGs, which are essential for achieving global health and international development. These results are relevant to understanding the direct relationship between SDH and the population’s health-disease process, integrating with the principles of the SUS and the intersectoral approach. Another relevant theme identified was hyperconnectivity and its possible threats to mental health, a global occurrence that urges us to reflect on strategies for dealing with constant exposure to the digital world.

Conducting the research using the Culture Circles proposed by Freire promoted critical dialog about the PHC context, enabling in-depth reflections on the development of health promotion strategies by managers.

It is recommended to carry out further studies regarding health promotion strategies in conjunction with the Social Determinants of Health, as this is essential for improving the work process of professionals at different levels of care.
