# Supplementary material for: Culture circle in primary care: dialogues with managers on health promotion
Source: Rev Esc Enferm USP. 2024 Jul 8;58:e20230420. doi: 10.1590/1980-220X-REEUSP-2023-0420en (PMC11236274; doi:10.1590/1980-220X-REEUSP-2023-0420en)
Supplement: Translation

# Translation: Círculo de cultura na atenção primária: diálogos com gestores sobre promoção da saúde

**DOI:** 10.1590/1980-220X-REEUSP-2023-0420pt

**Authors:** Ivonete Teresinha Schulter Buss Heidemann, Michelle Kuntz Durand, Izaltina Adão, Priscila Juceli Romanoski, Adriana Rufino Moreira, Aline Megumi Arakawa Belaunde, Vladimir Araujo da Silva, Kamila Soares Maciel

**Affiliations:** 1 Universidade Federal de Santa Catarina, Departamento de Enfermagem, Programa de Pós-Graduação em Enfermagem. Florianópolis, SC, Brasil.; 2 Universidade Federal de Santa Catarina, Departamento de Enfermagem. Florianópolis, SC, Brasil.; 3 Universidade Federal de Santa Catarina, Departamento de Saúde Pública, Programa de Pós-Graduação em Saúde Coletiva. Florianópolis, SC, Brasil.; 4 Universidade Federal de Santa Catarina, Departamento de Fonoaudiologia. Florianópolis, SC, Brasil.; 5 Universidade Federal de Santa Catarina, Centro de Ciências Rurais, Departamento de Biociências e Saúde Única. Santa Catarina, SC, Brasil.

**Keywords:** Promoção da Saúde, Sistema Único de Saúde, Atenção Primária à Saúde, Pesquisa Participativa Baseada na Comunidade, Determinantes Sociais da Saúde

## Abstract

**Objetivo:**

Identificar estratégias de promoção da saúde utilizadas por gestores na atenção primária à saúde.

**Método:**

Pesquisa qualitativa, de natureza ação participante, que adotou como referencial metodológico o Círculo de Cultura proposto por Paulo Freire. Participaram 11 gestores da atenção primária à saúde de um município de médio porte do sul do Brasil.

**Resultados:**

Surgiram nove temas geradores categorizados em quatro temáticas que evidenciam a interconexão entre promoção da saúde, determinantes sociais e atenção primária à saúde. Essas temáticas destacam abordagens preventivas, hábitos saudáveis e sublinham a necessidade de uma abordagem multidisciplinar na atenção à saúde, reconhecendo a complexidade das dimensões envolvidas, a influência dos determinantes sociais, questões ambientais e sanitárias. Tais aspectos demandam políticas e ações intersetoriais, evidenciando a viabilidade da promoção da saúde alinhada aos princípios do Sistema Único de Saúde.

**Considerações Finais:**

Destaca-se a autonomia dos profissionais que atuam em serviços de atenção primária à saúde, especialmente a do enfermeiro, que desempenha um papel central na conectividade e ordenação das ações de promoção da saúde.

## INTRODUÇÃO

A promoção da saúde constitui um conjunto de estratégias promissoras para abordar questões de saúde, tanto individual quanto coletivamente, visando aprimorar as condições de saúde. Caracteriza-se pela cooperação intrassetorial e intersetorial, estabelece conexões com redes de proteção social e promove ampla participação e controle social abrangente. Seu propósito é reduzir vulnerabilidades e riscos à saúde decorrentes de determinantes sociais, econômicos, políticos, culturais e ambientais. Considerando a concepção ampla do processo saúde-doença e de seus determinantes, essa estratégia busca integrar conhecimentos técnicos e populares. A promoção da saúde engloba ações voltadas para a mudança de comportamento individual, intervenções coletivas e políticas públicas integradas^(1)^.

A I Conferência Internacional sobre Cuidados Primários de Saúde, realizada em Alma-Ata, em 1978, trouxe contribuições para implementar a meta “saúde para todos no ano 2000”, com ênfase na proposta da Atenção Primária à Saúde (APS) como elemento central na organização de sistemas voltados à promoção da saúde universal^(2)^. Dentre as diversas Conferências Internacionais que dialogaram sobre a Promoção da Saúde, destaca-se a realizada em 1986, em Ottawa, no Canadá, quando os países se comprometeram a assumir ações promotoras da saúde para a condução de suas políticas. Nela foram recomendadas cinco estratégias básicas para a promoção da saúde: construir políticas públicas de saúde; criar ambientes de apoio; fortalecer a ação comunitária; desenvolver habilidades pessoais; e reorientar os serviços de saúde^(3)^. Por conseguinte, a IX Conferência de Shangai, de 2016, reforçou atividades de promoção da saúde, condizentes com a nova Agenda 2030 e os ODS^(1)^. A Declaração de Shanghai enfatiza a importância da autonomia das pessoas no controle de sua própria saúde, com a escolha de um estilo de vida saudável^(4)^. Os compromissos estabelecidos visam acelerar o financiamento para a promoção da saúde e a implementação dos ODS. Diversas propostas foram apresentadas para alcançar esse objetivo, incluindo a adoção de políticas que favoreçam mulheres e populações em vulnerabilidade social e ambiental, o reconhecimento do direito à saúde como um bem comum e fundamental para todos e a ênfase na importância de cidades e comunidades como ambientes essenciais para a promoção da saúde da população^(1)^.

Em 2018, a Conferência Global sobre Atenção Primária à Saúde em Astana buscou reafirmar o compromisso com a APS visando a Cobertura Universal de Saúde e os Objetivos do Desenvolvimento Sustentável (ODS). No entanto, a Cobertura Universal de Saúde, ao deslocar o direito à saúde fundamentado na igualdade para um princípio de cobertura financeiramente acessível, contraria o espírito de Alma-Ata e dos ODS, podendo resultar em disparidades. Críticos da Carta de Astana destacam essa discrepância, advogando pela APS integral e pelo direito universal à saúde global. No entanto, a Declaração de Alma-Ata mantém-se como uma referência internacional, superando à de Astana em princípios de justiça social e abordagem abrangente da APS^(5)^.

Entende-se que é necessário considerar as pessoas com hiperconectividade como parte da população vulnerável. Urgem políticas públicas para mitigar o sofrimento psíquico e, assim, promover a saúde mental da população assistida por nós, profissionais de saúde, visando alcançar a integralidade na atenção à saúde. Alinhada aos Determinantes Sociais da Saúde (DSS), a promoção da saúde subsidia o acesso à informação, amplia experiências e habilidades na vida das pessoas, possibilitando escolhas por um estilo de vida mais saudável. Essa abordagem visa reduzir as disparidades no estado de saúde da população, garantindo assistência e recursos de forma equitativa^(6)^.

A APS, ao ordenar o cuidado na rede de atenção à saúde, conduz as ações promotoras da saúde por meio de abordagens intersetoriais, englobando os setores da Saúde, Assistência Social e Educação^(6)^. Entretanto, para alcançar a equidade na saúde, torna-se crucial combater as desigualdades decorrentes de políticas e práticas históricas e atuais, incluindo os determinantes sociais e estruturais que resultam em exposição à insegurança alimentar, instabilidade habitacional e dificuldade de acesso a bens, serviços e oportunidades. Esse desafio é particularmente relevante quando se consideram pessoas em condições ou riscos de vulnerabilidade e exclusão social, associadas a fatores como raça, etnia, gênero, orientação sexual e imigração^(7)^. Diante desse contexto, este estudo tem como objetivo identificar as estratégias de promoção da saúde utilizadas pelos gestores na APS.

## MÉTODO

Trata-se de uma pesquisa qualitativa de natureza ação participante, que adotou como referencial teórico-metodológico o Itinerário de Pesquisa proposto por Paulo Freire, implementado por meio de Círculos de Cultura. Em um processo dialógico e dialético, promovem-se interações dinâmicas, críticas e horizontalizadas entre os participantes, suas experiências, conhecimentos e habilidades, visando à transformação política, social e educacional^(8)^. O Itinerário de Pesquisa abrange três momentos dialéticos e flexíveis, permitindo a utilização de elementos indutivos ou dedutivos nas etapas de investigação temática, codificação e descodificação, e desvelamento crítico^(9)^. A redação deste artigo segue o protocolo do *Consolidated Criteria for Reporting Qualitative Research* (COREQ)^(10)^.

O Círculo de Cultura foi realizado em um município com cerca de 40 mil habitantes, destacando-se nas áreas de agricultura, comércio e indústrias. A entrada no campo ocorreu após contato com um pesquisador representante de uma universidade local, estabelecendo uma conexão teórico-prática com a APS. Na ocasião, 11 gestores da APS participaram do evento. Este estudo abrangeu municípios da mesorregião de um estado do sul do Brasil, proporcionando resultados relevantes por meio da aplicação do método de pesquisa voltado para a temática da promoção da saúde. Os critérios de inclusão consideraram a condição de gestor e o consentimento para participar ativamente do círculo de cultura. Gestores afastados por férias ou licença durante a investigação temática foram excluídos. Para preservar o anonimato dos participantes, foram utilizados codinomes de flores escolhidos por eles.

A coleta de dados ocorreu por meio de um Círculo de Cultura, com duração total de duas horas, realizado em 11 de abril de 2023. Este encontro teve lugar no auditório da Secretaria Municipal de Saúde, coincidindo com a reunião mensal dos gestores municipais. Durante a reunião geral, foi reservado um período de duas horas para a realização do Círculo de Cultura, conforme previamente acordado com os participantes. Os mediadores responsáveis pela facilitação do Círculo de Cultura eram pesquisadores experientes, especializados e com profundo conhecimento no Itinerário de Pesquisa de Paulo Freire, utilizado neste estudo qualitativo. É relevante destacar que os mediadores mantiveram neutralidade, sem vínculos com os participantes, criando um ambiente propício para o diálogo, preservando a ética e o anonimato entre os envolvidos.

No dia da realização do Círculo de Cultura, foram apresentados inicialmente os objetivos do estudo e a metodologia a ser adotada. Em seguida, os participantes participaram de uma dinâmica de apresentação e preenchimento de dados socioeconômicos. Posteriormente, teve início a fase inicial do estudo, conhecida como investigação temática. Dentro do contexto do Itinerário de Pesquisa de Paulo Freire, não é comum fazer referência explícita a um “codebook” ou “roteiro” no sentido tradicional, uma vez que esse método favorece a construção coletiva do conhecimento e a flexibilidade no processo de investigação.

Os participantes foram divididos em três subgrupos, nos quais foram incentivados a dialogar e registrar os temas geradores em cartolinas coloridas, respondendo à questão orientadora: “Quais estratégias de promoção da saúde são abordadas na APS? Identifique as potencialidades e os desafios associados ao trabalho na APS.” Após a etapa de investigação, os subgrupos reuniram-se novamente em um grande grupo e compartilharam suas descobertas temáticas. Nove temas geradores foram identificados e posteriormente agrupados em quatro temáticas de relevância significativa: 1) Educação em saúde: promoção da saúde por meio da educação em saúde; grupos de educação em saúde; Programa Saúde na Escola (PSE): estratégias/práticas de promoção da saúde; Práticas Integrativas e Complementares em Saúde (PICS). 2) Relação entre os DSS e a situação de saúde-doença: DSS e educação permanente; DSS e a situação de saúde-doença dos “usuários”. 3) Princípios do Sistema Único de Saúde (SUS) e a intersetorialidade. 4) Conectividade e relação com a saúde mental: dependência tecnológica e conectividade; redes sociais e promoção da saúde mental.

Para fomentar o diálogo e torná-lo mais dinâmico, interativo e concreto, os participantes optaram pela analogia de uma flor para representar as etapas do Itinerário de Pesquisa de Paulo Freire e o contexto de atuação como gestores em uma cidade do meio oeste de Santa Catarina, conforme a Figura 1. Nessa analogia, refletiu-se sobre as partes interligadas de uma flor, analogamente ao Itinerário de Pesquisa de Paulo Freire.

**Figura 1:** Itinerário de Pesquisa: analogia com a flor.

Fonte: elaborado pelos autores.

Na segunda etapa, denominada codificação e descodificação, discutiram-se as quatro temáticas emergentes no grupo principal. Os mediadores engajaram-se em diálogos com os participantes, incentivando-os a contemplar sua realidade e refletir sobre a promoção da saúde e os DSS.

Na etapa do desvelamento crítico, aprofundou-se a discussão dos temas delineados nas fases anteriores. Com o consentimento dos participantes, os diálogos foram gravados por aproximadamente quatro horas utilizando gravadores de áudio para capturar as discussões. As principais temáticas abordadas foram registradas em um caderno de campo e posteriormente transcritas para uma análise detalhada.

A análise dos dados foi conduzida simultaneamente ao desenvolvimento do Círculo de Cultura, alinhando-se aos princípios do Itinerário de Pesquisa de Paulo Freire^(9)^. Este processo analítico contínuo foi realizado em interação com todos os participantes do estudo^(8)^, gestores e mediadores, sem a necessidade do emprego de software de apoio às análises de dados qualitativos.

Para atender aos aspectos éticos inerentes à pesquisa com seres humanos, os pesquisadores seguiram as recomendações das Resoluções 466/12 do Conselho Nacional de Saúde e suas complementares^(11)^.

## RESULTADOS

Foram explorados nove temas geradores categorizados em quatro temáticas: 1) Educação em saúde; 2) Relação entre os DSS e a situação de saúde-doença; 3) Princípios do SUS e a intersetorialidade; 4) Conectividade e a relação com a saúde mental.

### 1. Educação em Saúde

A educação em saúde foi amplamente discutida, sendo os participantes unânimes ao considerá-la como um elemento fundamental para a promoção da saúde. O debate centrou-se na importância das orientações de saúde e na necessidade de conscientização da população em relação à educação em saúde. Foram abordadas ações voltadas à promoção da saúde, prevenção de doenças e a importância de envolver grupos de apoio e a comunidade em geral na disseminação de informações relacionadas à saúde. Destacou-se a relevância de uma abordagem multidisciplinar, reconhecendo que um único profissional não pode abranger todas as facetas da saúde.

*As equipes multiprofissionais são importantes para auxiliar à comunidade e reforçar a saúde como um todo. Nosso trabalho busca a coletividade e a organização para oferecer atendimento de qualidade em cada unidade* (Violeta).

Destacaram como pontos fortes no município o princípio da integralidade, ressaltando que este princípio *orienta nossa abordagem para atingir diversos públicos por meio de diferentes estratégias* (Flor de Lótus).

Os grupos terapêuticos abrangem diversas condições de saúde, como gestantes, hipertensos, diabéticos e tabagistas. Por outro lado, o PSE estabelece parcerias com a secretaria de educação e promove intervenções desde a infância até o ensino médio, abordando diversas temáticas. Como exemplo, destaca-se o projeto ‘*Vida Saudável’, resultado de uma parceria entre as secretarias de saúde e esportes, que oferece aulas de dança para diferentes faixas etárias, e promove não apenas a saúde física, mas também a satisfação e o bem-estar dos participantes* (Violeta).

Outro ponto-chave destacado é a influência da cultura na promoção da saúde. A cultura desempenha um papel fundamental na formação dos hábitos de vida, nas atitudes em relação à saúde e na maneira como as pessoas buscam cuidados de saúde. Mencionaram *em muitas culturas, o foco principal pode estar na assistência médica curativa, ou seja, tratar doenças após sua manifestação, em vez de priorizar a prevenção* (Tulipa).

*A ênfase na parte curativa pode ser um reflexo das crenças e valores culturais de uma comunidade. A busca por assistência médica pode ser vista como uma demonstração de cuidado e responsabilidade, enquanto a promoção da saúde, que se concentra muitas vezes na prevenção, pode ser menos enfatizada* (Orquídea).

A promoção da saúde também foi discutida no grupo, no sentido de compreensão de que as práticas integrativas e complementares em saúde são importantes para a implantação e implementação da promoção da saúde no território.

*Nós temos hoje dentro desse contexto aqui, a auriculoterapia, reiki, e temos colegas aqui que são terapeutas e que tentam associar isso dentro da promoção em saúde. Hoje sabemos que essa busca tanto da relação da promoção em saúde voltada para a área espiritual, os conceitos de uma fala direcionada, de um acolhimento caprichado, de um ambiente onde se possa trabalhar a promoção em saúde* (Arruda).

### 2. Relação Entre os DSS e a Situação de Saúde-Doença

Os participantes reconheceram a presença dos DSS que não estão sob controle direto dos profissionais de saúde, tais como questões ambientais, poluição de empresas e más condições sanitárias. Esses fatores podem influenciar o surgimento de doenças e impactar o bem-estar da comunidade, mas ultrapassam a gestão das unidades de saúde, dependendo de ações e medidas em âmbito municipal, estadual e nacional. Os participantes ressaltaram que *os profissionais de saúde podem desempenhar um papel na prevenção e no tratamento de doenças, mas a resolução dessas questões requer esforços coletivos em níveis superiores* (Rosa).

Discutiram a relevância da orientação da população sobre determinantes sociais passíveis de conscientização, como estilo de vida e condições de higiene. Abordaram os desafios enfrentados na comunicação efetiva e compreensão nas intervenções de saúde. Também refletiram sobre a necessidade de capacitação dos profissionais de saúde e as limitações de tempo para promoção da saúde, dadas as demandas diárias que tendem a dificultar a abrangência necessária.

### 3. Princípios do SUS e a Intersetorialidade

Os profissionais da APS destacaram a aplicabilidade da promoção da saúde ancorada nos princípios do SUS: universalidade, equidade, integralidade, descentralização e participação da população. Evidenciaram a implementação de ações direcionadas a grupos, ressaltando a importância da estratificação da população para uma atenção mais efetiva à saúde, com enfoque nas condições crônicas.

*Quando falamos em promoção, buscamos atingir todos os públicos e de diversas maneiras. [...] cada unidade trabalha com a sua equipe, sua área, mas sempre buscando abranger grupos de gestantes, hipertensos, diabéticos, tabagistas, insulinodependentes...* (Flor de Lótus).

A intersetorialidade, evidenciada nas declarações, ressalta a necessidade de associação e envolvimento de profissionais de diversas áreas e secretarias para a eficácia das ações de promoção da saúde.

*Conforme o calendário, a gente trabalha todas as temáticas e todos os eixos. Às vezes também fugimos um pouquinho conforme a solicitação da secretaria, quando eles acham necessário abordar algum tema que naquele momento está sendo mais relevante. [...] o projeto ‘Vida Saudável’ trabalha nos bairros! É uma parceria da secretaria de esportes com a secretaria de saúde, que oferece aulas de dança para todos os níveis de idade. Tem uma abrangência bem legal e uma participação bem boa!* (Flor de Lótus).

Nesse contexto, destaca-se a importância da autonomia dos profissionais, especialmente do enfermeiro, que desempenha um papel central na coordenação e integração das ações de promoção da saúde, *... na organização da demanda das unidades, para conseguir atender da melhor forma possível o nosso público* (Flor de Lótus).

### 4. Conectividade e a Relação Com a Saúde Mental

A tecnologia foi destacada como um determinante a ser considerado na avaliação das causas do adoecimento mental em todas as faixas etárias, alertando para a ausência de protocolos clínicos que auxiliem no enfrentamento das novas demandas emergentes.

*Nós estamos tendo hoje, não só adolescentes, mas também adultos e idosos que não têm muito o que fazer, que ficam nas redes sociais recebendo informações que estão trazendo um agravo psíquico muito grande, uma grande dependência. E nós não estamos sabendo fazer esse manejo com essas pessoas* (Arruda).

Os profissionais evidenciaram conhecimento e inquietação em relação à sua preparação para lidar com doenças decorrentes da exposição excessiva à tecnologia, ao tempo prolongado diante das telas e à precoce exposição das crianças à conectividade. Tais fatores podem ocasionar danos não apenas à saúde mental dos usuários, mas também às relações familiares e sociais. Isso destaca a importância da educação e conscientização da população sobre essas questões.

*Nós vamos ter que saber manejar, como vamos acolher e não só acolher a pessoa com dependência tecnológica, mas também saber acolher a família. [...] a dependência está tão grande... A maioria aqui tem filhos. Tu vais num restaurante, a criança está chorando, a mãe fica no celular e a criança comendo e olhando um filminho* (Arruda).

Também expressaram preocupação diante da ausência de políticas públicas direcionadas à publicidade e propaganda em redes sociais, especialmente no que se refere a ações de promoção da saúde, com foco na saúde mental.

*A gente está vivendo uma pandemia muito grande da dependência química [...] se a gente olhar hoje na TV quantos comerciais você vê falando contra as drogas? Nenhum! Você só vê propaganda de cerveja! É preciso entender melhor o que é o crack e isso você também não vê!* (Arruda).

No diálogo com os participantes, identificou-se uma forte relação entre a conectividade e uma vulnerabilidade vivenciada, associada a um aumento de ansiedade e estresse, bem como à dificuldade de conexão com os recursos tecnológicos.

## DISCUSSÃO

Os resultados revelam que as estratégias de promoção da saúde empregadas por gestores na APS contemplam, ou indicam preocupação em abranger, as cinco estratégias recomendadas na Carta de Ottawa^(3)^. Além de evidenciar a educação em saúde como elemento fundamental, identificam também questões que podem contribuir para a implementação de estratégias não abordadas, promovendo a equidade na saúde^(7)^ e alcançando os ODS^(12)^ fundamentais para a saúde global e o desenvolvimento internacional.

### 1. Educação em Saúde

A educação em saúde é percebida como uma das estratégias de promoção da saúde, representando amplas abordagens com o objetivo de aprimorar as condições de vida individuais e coletivas. Além das considerações clínicas, englobam ações ampliadas de prevenção de doenças, promoção de um estilo de vida saudável, com ênfase na prática de atividade física, alimentação balanceada, redução do estresse, promoção da saúde mental, acesso a serviços de saúde e educação em saúde, entre outras^(1,2)^.

A orientação e conscientização da população configuram-se como uma oportunidade para ampliar o conhecimento e compreensão dos educandos sobre sua própria realidade^(13)^. As intervenções educativas em saúde, ao serem direcionadas a grupos específicos, têm o potencial de provocar mudanças significativas no nível de conhecimento e atitudes, favorecendo a modificação de hábitos e comportamentos^(14)^. Nesse contexto, a educação em saúde é considerada um dos principais eixos estratégicos para a promoção da saúde, com a capacidade de transformar as condições de vida e saúde das pessoas^(15)^. De fato, esse empoderamento e protagonismo, inerentes à educação em saúde, representam a estratégia para “fortalecer a ação comunitária” na APS, conforme recomendado pela Carta de Ottawa^(3)^. promovendo autonomia e o compartilhamento de experiências em busca da qualidade de vida^(15)^. No entanto, para alcançar impactos nas dimensões individual, social e ambiental, torna-se crucial envolver uma equipe multidisciplinar^(14)^.

A atuação dos profissionais vai além da orientação e transmissão de informações, sendo essencial incorporar abordagens interdisciplinares, planejamento, conteúdos e métodos adequados a realidade local. Com efeito, o Projeto *“Vida Saudável”*, referido pelos gestores e elaborado com base nos pressupostos supracitados, está alinhado à estratégia “criar ambientes de apoio” recomendada na Carta de Ottawa^(3)^. Tal abordagem possibilita a reorientação e a integralidade dos cuidados de saúde. É imperativo que essa educação seja embasada nos princípios propostos por Paulo Freire, caracterizando-se como uma inserção crítica na realidade, permeada por diálogo, reflexão, comunicação, práticas colaborativas e reciprocidade entre os envolvidos. Isso propicia a construção de novos saberes e a transformação tanto do indivíduo quanto do mundo^(16)^.

Em se tratando de intersetorialidade, o PSE é uma estratégia significativa em promoção da saúde capaz de ampliar parcerias intersetoriais e oferecer intervenções adaptadas a diferentes níveis de escolaridade, abrangendo diversas temáticas adequadas a realidade local. De fato, o programa fortalece a relação entre as redes de educação e de saúde, entretanto, foram necessários muitos diversos até que os profissionais de ambas as áreas pudessem compreender plenamente a importância da atuação intersetorial^(17)^. Ao aproximar os serviços de saúde das escolas, o PSE permite uma intervenção mais abrangente, contribuindo diretamente para a formação de pessoas mais conscientes e saudáveis.

Ainda, observa-se o potencial significativo das PICS como uma estratégia promissora para a promoção da saúde e a integralidade do cuidado. Atualmente, a PNPIC reconhece 29 modalidades de terapias baseadas em saberes e práticas não convencionais de saúde^(18)^, as quais têm ganhado crescente popularidade em virtude de seus benefícios, crenças e valores. Esse reconhecimento converge com a influência da cultura na promoção da saúde, uma vez que possibilita a compreensão mais profunda dos hábitos, das atitudes e da forma como que as pessoas buscam cuidados em saúde.

### 2. Relação Entre os DSS e a Situação de Saúde-Doença

A concepção ampliada do processo saúde-doença parte do pressuposto de que os DSS – condições humanas relacionadas à alimentação, saneamento, habitação, trabalho, renda, acesso à educação e serviços de saúde, transporte, ecossistema estável e recursos sustentáveis – influenciam o grau de susceptibilidade a fatores de risco e a ocorrência de problemas de saúde na população^(19)^. Com efeito, um estudo realizado com 27 profissionais da saúde, incluindo 18 educadores, identificou que condições de vida desfavoráveis, hábitos pouco saudáveis e relações sociais frágeis tendem a influenciar negativamente o acompanhamento regular do crescimento e desenvolvimento, assim como o desempenho escolar de crianças^(20)^.

Fundamentados nessa concepção, os profissionais destacaram a importância de conscientizar a população sobre a adoção de um estilo de vida saudável e orientar sobre a higienização. Durante o Círculo de Cultura, enfatizaram os fatores comportamentais, ambientais/sanitários, econômicos e sociais, em detrimento dos fatores psicológicos inerentes à situação de saúde-doença.

Não obstante, torna-se imprescindível considerar a interação das forças pessoais, comunitárias e sociais na conservação das condições ambientais e de saúde individuais e coletivas. Aceitar que a situação de saúde seja determinada significa subestimar a potencialidade da natureza, criatividade e autonomia das pessoas e da sociedade, e sua capacidade de se auto-organizar, agir e modificar ou desconstruir o que foi historicamente construído pela ação humana^(21)^.

Talvez por não terem clareza sobre a relevância de considerar os aspectos supracitados, os profissionais reconhecem que questões ambientais, como a poluição produzida pelas empresas e más condições sanitárias, escapam de sua gerência. No entanto, a percepção dos gestores de que a resolução dessas questões requer esforços coletivos em níveis superiores, especialmente a crítica à inexistência de publicidade e propaganda na TV para o enfrentamento do uso de drogas ilícitas e para a promoção de saúde mental, sugere e/ou pode subsidiar a estratégia “construir políticas públicas de saúde” recomendada na Carta de Ottawa^(3)^.

### 3. Princípios do SUS e a Intersetorialidade

Os DSS são condições que moldam o processo de saúde e doença. Atualmente, além dos determinantes proximais relacionados ao estilo de vida e distais de renda, educação, ocupação, estrutura familiar, disponibilidade de serviços de saúde, apoio social, discriminação racial, até então já conhecidos é preciso refletir para fatores ambientais, a saúde digital, migração, determinantes comerciais, entre outros^(22)^. Em nosso estudo, os profissionais reconhecem os impactos dos DSS e destacam que a educação é promissora no enfrentamento das condições crônicas, fornecendo informações globais e apoio ao autocuidado.

Considerando as desigualdades socioeconômicas no Brasil e o estilo de vida como determinantes de saúde, um estudo com dados da Pesquisa Nacional de Saúde de 2013 destacou a necessidade de monitorar essas disparidades para orientar políticas públicas. Identificou-se que as desigualdades são mais prevalentes em pessoas com menor escolaridade, não brancas e sem planos de saúde privados. O sexo masculino apresenta maiores índices de tabagismo, consumo de álcool e carnes gordurosas, com menor ingestão de frutas, legumes e verduras^(23)^.

Mundialmente, a redução das Condições Crônicas Não Transmissíveis (CCNT) são essenciais no contexto do desenvolvimento global, alinhando-se aos ODS com a meta de reduzir a mortalidade prematura em um terço até 2030. Este estudo destaca a interligação dessas metas com outros nove objetivos, ressaltando a necessidade de estratégias para ganhos econômicos e o planejamento de cidades sustentáveis^(24)^. Os maiores riscos de mortalidade por CCNT são observados em países de baixa e média renda, enfatizando a importância de abordagens intersetoriais que abranjam metas como redução da pobreza, combate à fome, educação, igualdade de gênero, crescimento econômico, equidade, vida em cidades e produção e consumo sustentável^(24)^.

Em um estudo na Inglaterra (2016–2017) com crianças de 10 a 11 anos, observou-se uma prevalência de obesidade de 26% em regiões mais carentes e 11% em regiões menos pobres. Destaca-se que a desigualdade na obesidade infantil persiste na idade adulta, resultando em uma crescente disparidade nos problemas de saúde associados à obesidade^(25)^.

A emergência climática, a epidemia de doenças não transmissíveis e o impacto significativo de setores específicos (tabaco, alimentos ultraprocessados, combustíveis fósseis e álcool) revelam a magnitude e os altos custos econômicos associados a esses desafios^(26)^. Estudo que buscou definir e conceituar os determinantes comerciais da saúde ressalta que, embora os determinantes comerciais possam ter impactos positivos, há evidências substanciais de que certas práticas e produtos, especialmente de grandes empresas transnacionais, contribuem para problemas de saúde, danos ambientais e desigualdades sociais e de saúde evitáveis. Os determinantes comerciais necessitam de atenção especial, pois muitas vezes ignora políticas públicas em prol do lucro, enfraquecendo governos e Estado. Esse estudo enfatiza ainda a urgência de implementar regulamentações alimentares efetivas pelo Estado^(26)^.

Os DSS moldam quatro fatores de risco comportamental significativos: alimentação inadequada, falta de atividade física, tabagismo e consumo excessivo de álcool, além de três condições patológicas - hipertensão arterial, diabetes e obesidade^(25)^. Nossa pesquisa evidencia a presença cotidiana de práticas educativas em grupo, promovendo os princípios do SUS.

A integralidade, entendida como o leque dos serviços que a APS pode oferecer ao usuário, constitui a essência da APS, sendo o eixo central para a operacionalização dos demais atributos. Entretanto, uma avaliação da integralidade da APS em diferentes países, realizada por meio de revisão sistemática com o *Primary Care Assessment Tool* (PCATool), indica que o baixo grau de orientação da APS para a integralidade dos serviços disponíveis pode ser interpretado como uma falta de compreensão das reais demandas dos usuários, requerendo ações concretas para fortalecer a APS como base dos sistemas de saúde^(27)^.

No que tange à intersetorialidade, inerente ao Projeto “Vida Saudável”, apresentado pelos gestores, incorpora a intersetorialidade ao unir as secretarias de saúde e esporte, estratificando a população em grupos como gestantes, hipertensos, diabéticos e tabagistas, seguindo a estratégia de “reorientar os serviços de saúde” preconizada na Carta de Ottawa^(3)^. Entretanto, a crítica à cultura centrada na assistência médica curativa não é acompanhada por propostas concretas para enfrentar o problema identificado. Adicionalmente, a ausência de menção a estratégias específicas para grupos vulneráveis^(7)^ sugere resistência ou dificuldade dos gestores em lidar com esses públicos, comprometendo o alcance da equidade na saúde.

### 4. A Conectividade e a Relação Com a Saúde Mental

Em nossa pesquisa, também destacamos preocupações em relação à conectividade e sua relação com a saúde mental, um determinante que tem sido cada vez mais objeto de estudo em contextos de condições crônicas. Segundo a OMS, as condições de saúde mental respondem por 16% da carga global de doenças e lesões em pessoas entre 10 e 19 anos. A depressão figura como uma das principais causas de doença e incapacidade entre adolescentes, enquanto o suicídio se posiciona como a terceira principal causa de morte nessa faixa etária (15 a 19 anos). As repercussões de negligenciar a saúde mental dos adolescentes extrapolam a adolescência, afetando adversamente a saúde física e mental e restringindo oportunidades futuras. O relatório também destaca que a influência da mídia e as normas de gênero podem amplificar a disparidade entre a realidade vivida por um adolescente e suas percepções ou aspirações para o futuro^(28)^.

Na contemporaneidade, enfrentamos uma avalanche de informações impulsionada pelo uso da internet e das redes sociais. Embora essas ferramentas facilitem diversas atividades cotidianas, sua presença constante pode ter efeitos adversos, desviando nossa atenção e foco quando estamos com pessoas significativas, como nossos familiares. Este fenômeno global suscita reflexões sobre a gestão adequada da hiperconectividade, considerando seu impacto na saúde mental. A hiperconectividade^(29)^, abrange todas as formas de comunicação na rede, envolvendo interações entre pessoas, de pessoa para máquina e entre máquinas. Refere-se à constante conexão a dispositivos tecnológicos, como smartphones, notebooks e computadores.

Na rede, podemos nos reinventar; nesse mundo onde valorizamos mais o TER do que o SER, sujeitamo-nos a autocoerções que podem desencadear transformações em nossa forma de viver e sofrer. Nas redes sociais, apresentamos o que queremos ser, não necessariamente quem somos verdadeiramente. A revolução digital, com sua aguda hiperconexão, suscita discussões relevantes sobre nossa liberdade de expressão, submetendo-nos a uma ditadura da melhor performance, onde mostramos apenas o lado positivo e, se necessário, inventamos. Nesse contexto, é crucial considerar os impactos nos sofrimentos psíquicos e narcísicos, pois surgem novas dinâmicas em relações amorosas, trabalho e interações pessoais, alterando o psiquismo diante dessa nova realidade^(30)^.

Uma das principais preocupações dos profissionais, conforme evidenciado no estudo, é o manejo das numerosas informações proporcionadas pela tecnologia aos usuários. A lacuna na formação contínua dos profissionais e a subsequente dificuldade em lidar com as novas demandas emergentes, conforme apontado pelos gestores, ressaltam a importância e a carência da implementação da estratégia “desenvolver habilidades pessoais”, recomendada na Carta de Ottawa^(3)^.

Entre as limitações deste estudo, destaca-se o desafio de encontrar um horário que fosse conveniente para a participação de todos os profissionais e gestores no Círculo de Cultura, considerando a carga de atividades inerentes ao seu processo de trabalho.

## Considerações Finais

Este estudo evidenciou que as estratégias de promoção da saúde adotadas por gestores da APS estão conectadas com as cinco estratégias recomendadas na Carta de Ottawa ou podem subsidiar o alcance das estratégias não contempladas, da equidade na saúde e dos ODS, essenciais para o alcance da saúde global e do desenvolvimento internacional. Esses resultados são relevantes para compreender a relação direta dos DSS com o processo de saúde-doença da população, integrando-se aos princípios do SUS e à abordagem intersetorial. Outra temática relevante identificada foi a hiperconectividade e suas possíveis ameaças à saúde mental, uma ocorrência global que nos instiga a refletir sobre estratégias para lidar com a constante exposição ao mundo digital.

A condução da pesquisa por meio dos Círculos de Cultura propostos por Freire promoveu diálogos críticos sobre o contexto da APS, possibilitando reflexões aprofundadas sobre o desenvolvimento das estratégias de promoção da saúde pelos gestores.

Recomenda-se a realização de novos estudos que abordem as estratégias de promoção da saúde em articulação com os Determinantes Sociais da Saúde, pois isso é essencial para aprimorar o processo de trabalho dos profissionais em diferentes níveis de atenção.
